# Williams syndrome: reduced orienting to other’s eyes in a hypersocial phenotype

**DOI:** 10.1007/s10803-022-05563-6

**Published:** 2022-04-20

**Authors:** Johan Lundin Kleberg, Deborah Riby, Christine Fawcett, Hanna Björlin Avdic, Matilda A. Frick, Karin C. Brocki, Jens Högström, Eva Serlachius, Ann Nordgren, Charlotte Willfors

**Affiliations:** 1 Centre for Psychiatry Research, Department of Clinical Neuroscience, Karolinska Institute and Stockholm Health Care Services, Region Stockholm, Sweden; 2 Department of Molecular Medicine and Surgery, Karolinska Institute, Stockholm, Sweden; 3 Centre for Developmental Disorders, Department of Psychology, Durham University, Durham, United Kingdom; 4 Department of Psychology, Uppsala University, Uppsala, Sweden; 5 Department of Medical Sciences, Uppsala University, Uppsala, Sweden; 6 Department of Clinical genetics, Karolinska University Hospital, Stockholm, Sweden

**Keywords:** Williams syndrome, Attention to eyes, Social motivation, Arousal, Eye tracking, Social orienting

## Abstract

Williams syndrome (WS) is a rare genetic condition associated with high sociability, intellectual disability, and social cognitive challenges. Attention to others’ eyes is crucial for social understanding. Orienting to, and from other’s eyes was studied in WS (n = 37, mean age = 23, age range 9–53). The WS group was compared to a typically developing comparison participants (n = 167) in stratified age groups from infancy to adulthood. Typically developing children and adults were quicker and more likely to orient to eyes than the mouth. This bias was absent in WS. The WS group had reduced peak saccadic velocities, indicating hypo-arousal. The current study indicates reduced orienting to others’ eyes in WS, which may affect social interaction skills.

Williams syndrome (WS) is a rare genetic condition occurring in around 1 in 7500 live births caused by a deletion of ~ 25–28 genes at chromosome 7q11.23 (Stromme, Bjornstad, & Ramstad, 2002). WS is characterized by a heightened probability of intellectual disability, short stature, cardiovascular anomalies, distinctive facial appearance, joint laxity, and other connective tissue abnormalities. The syndrome is also often associated with a personality characterized as “hyper-social” (Jones et al., 2000). For example, although there are vast individual differences, many individuals with WS are described as overly friendly and likely to approach strangers, showing excessive social interaction and being highly empathetic (Järvinen, Korenberg, & Bellugi, 2013; Jones et al., 2000). Observational studies show that WS individuals preferentially direct their attention to social stimuli such as faces and voices, although there is considerable individual variability (Doherty-Sneddon, Riby, Calderwood, & Ainsworth, 2009; Porter, Shaw, & Marsh, 2010; Riby & Hancock, 2008). Increased attention to faces has been reported in toddlerhood, and continues throughout development (D’Souza et al., 2015; Jones et al., 2000; Mervis et al., 2003).

Enhanced social attention in WS comes together with relative strengths in some areas of social perception (Jones et al., 2000; Sampaio et al., 2018; Vivanti, Hamner, & Lee, 2018). These strengths are particularly striking since WS is also associated with mild to moderate intellectual disability and particular challenges in areas such as visuospatial reasoning, memory and attention (Atkinson et al., 2007; Gregory et al., 2019; Miezah et al., 2020; Sampaio, Sousa, Férnandez, Henriques, & Gonçalves, 2008). WS has been a focus of interest for researchers from many disciplines, since this uneven cognitive profile indicates a dissociation between the social brain and mechanisms involved in domain-general learning (Barak & Feng, 2016; Sampaio et al., 2018). Genes commonly deleted in WS have been linked to sociability in the general human population (Barak & Feng, 2016; Crespi & Procyshyn, 2017; Zanella et al., 2019).

Deletions at the WS locus are associated with altered functioning of a number of neural systems important for social functioning, including the oxytocin system, the orbitofrontal cortex and the corpus callosum (Avery, Thornton-Wells, Anderson, & Blackford, 2012; Barak & Feng, 2016; Fan et al., 2017). WS is also associated with both structural and functional alterations within the amygdala (Lew et al., 2018) and altered connectivity between the amygdala and the visual and orbitofrontal cortex, areas implicated in social perception (Avery et al., 2012; Nir & Barak, 2020). Recently, studies of induced pluripotent stem cells (iPSCs) derived from individuals with WS have shown increased apoptosis, morphologic alterations including longer total dendrites and increasing dendritic spine number and an increase in calcium transient frequency and synchronized activity likely due to increased number of dendritic spines and synapses (Chailangkarn & Muotri, 2017).

WS has often been compared and contrasted with autism spectrum conditions (ASC), which are typically associated with social motivation differences, but relatively proficient visuospatial cognitive abilities (Barak & Feng, 2016). However, despite a propensity for high level of sociability, many individuals with WS face multiple challenges related to social interaction, which sometimes overlap with those seen in ASC. For example, they often have difficulties understanding others’ intentions (Van Herwegen, Smith, & Dimitriou, 2015) or gaze direction (Mobbs et al., 2004; Vivanti, Fanning, Hocking, Sievers, & Dissanayake, 2017). WS is also associated with a face-processing style based on individual features rather than configural properties (Deruelle, Mancini, Livet, Cassé-Perrot, & De Schonen, 1999; Karmiloff-Smith et al., 2004; Pavlova, Heiz, Sokolov, & Barisnikov, 2016); but see Tager-Flusberg, Plesa-Skwerer, Faja, & Joseph, 2003 who reported evidence of neurotypical holistic face processing patterns in WS). The wide range of difficulties with face processing in WS may be surprising, given the tendency for high sociability and an interest in faces. A possible explanation for this apparent contradiction is that they fail to process information within certain highly informative facial regions, such as the eyes.

The eye region is arguably the most informative part of others faces for detecting information such as emotional states and intentions. Consistent with this, typically developing humans allocate the major part of their gaze to the eye region during face viewing (Lewkowicz & Hansen-Tift, 2012), and perceived eye contact is typically experienced as rewarding (Hietanen, 2018). A bias to attend to eyes is observed during the earliest stages of visual attention. In typical development, eyes attract quick and automatic gaze shifts from infancy and onwards (Johnson, Senju, & Tomalski, 2015; Kleberg, del Bianco, & Falck-Ytter, 2019), a process believed to be supported by a specialized brain network including the amygdala (Johnson et al., 2015; Spezio, Huang, Castelli, & Adolphs, 2007), a brain region with altered functional and structural properties in WS (Avery et al., 2012; Lew et al., 2018). Altered eye contact is observed already during the earliest time stages of processing in conditions affecting social interaction such as social anxiety disorder (Kleberg, Högström, Sundström, Frick, & Serlachius, 2021b) and autism (Hadjikhani et al., 2017). To understand eye contact processing in WS, it is therefore important to use methods with high temporal resolution, such as eye tracking.

Previous studies of eye contact in WS indicate an atypical profile, though its details remain unclear. A number of studies have suggested that some individuals with WS attend longer to other’s eyes than their typically developing peers do, indicated by observational (Jones et al., 2000), as well as experimental studies (Porter et al., 2010; Riby & Hancock, 2008). At the same time, unusual responses to eye contact have been reported. For example, using the Autism Diagnostic Observation Schedule (ADOS; Lord, Rutter, DiLavore, & Risi, 1999), Klein-Tasman et al. (2009) reported unusual eye contact in 50% of a group of young children (2.5–5.5 years) with WS. Unusual eye contact as operationalized in the ADOS includes behaviors such as avoidant as well as overly increased eye contact or poor integration of eye contact with other communicative behaviors. It is therefore not known from previous studies whether the previously observed pattern of increased attention to faces in WS is also found for the eye region specifically.

Why would individuals with WS look at others’ eyes for an extended time? At least three, not mutually exclusive, explanations are possible. First, WS may be associated with domain-general difficulties in attention disengagement (Breckenridge, Braddick, Anker, Woodhouse, & Atkinson, 2013) which could lead to prolonged gaze to eyes once the eyes are fixated (Porter et al., 2010; Riby & Hancock, 2008). Secondly, individuals with WS could be quicker to detect and orient to others’ eyes, although not necessarily slower in reorienting. Only one study has directly tested these first two hypotheses (Porter et al., 2010). In this small study (*n =* 16), on average, individuals with WS took longer to disengage their gaze from others’ eyes once they were fixated. In contrast, no evidence for quicker orienting to the eyes was found, contradicting the second hypothesis. Other studies examining attention to whole faces (but not eyes specifically) have also reported evidence for delayed disengagement rather than quicker orienting (Dodd & Porter, 2010; McGrath et al., 2016; Riby & Hancock, 2009), but have not been able to distinguish between these two processes in relation to the eye region specifically.

A third possible explanation is that increased gaze to eyes in WS is due to reduced physiological arousal (Doherty-Sneddon et al., 2009; Riby, Whittle, & Doherty-Sneddon, 2012). Studies in neurotypical populations have shown that direct gaze increases arousal (Hietanen, 2018), and that gaze aversion can serve as a means of arousal regulation (Doherty-Sneddon, Riby, & Whittle, 2012). Previous studies have hypothesized that individuals with WS may be able to hold eye contact for longer periods because lower arousal in this population reduces the need for gaze avoidance (Doherty-Sneddon et al., 2009). The first aim of the current study was to examine orienting to, and disengagement from, the eyes in individuals with WS. Secondly, we tested the hypothesis that increased attention to the eye region by individuals with WS is associated with hypo-arousal. Eye tracking was used to enable spatially and temporally precise measurements of visual attention.

Selection of appropriate comparison groups in perception research with participants with WS and other rare genetic conditions associated with intellectual disability is theoretically challenging. One option is to select a typically developing comparison group matched for chronological age. However, in this case, the comparison and study groups will differ on general intellectual ability, which may be a confounding factor in perception. Another option is to select a typically developing comparison group matched on performance on standardized IQ tests. This will typically result in a comparison group that is much younger than the study population, and therefore differing on multiple parameters. A third option is to compare the study group to a population with intellectual disability without a known genetic cause. Although this research design has several merits, “idiopathic” intellectual disability in the severe to medium range is in many cases likely to be associated with genetic alterations that are either undiagnosed or yet not described in the literature. Due to increased availability of whole-genome sequencing, knowledge about the genetic origins of intellectual disability is growing rapidly. Today, it is known that intellectual disability is of genetic origin in the majority of cases, and the number of identified conditions is increasing rapidly (Kvarnung & Nordgren, 2017). Comparison groups with idiopathic intellectual disability are therefore likely to be highly heterogeneous at a biological level, which limits the conclusions that can be drawn.

To avoid some of these shortcomings, we compared individuals with WS to a large typically developing comparison group (n = 167) covering a wide age range from 7 months to adulthood which was stratified into four different age groups (*see Participants*). This allowed us to compare behavior of the WS groups to typically developing individuals at different intellectual levels and developmental stages, as well as to map the developmental trajectory of the task in a typically developing sample. Sample sizes in previous eye tracking studies of WS have been small, typically including fewer than 20 participants, which limits conclusions that can be drawn or any consideration of within-syndrome variability. To avoid this shortcoming, we recruited a relatively large group of participants with WS (*n =* 41 tested; *n* = 37 in the final sample) in a broad age range (9–53 years).

*Hypotheses*.

We hypothesized that individuals with WS would be more likely and quicker to orient to eyes than typically developing individuals (*hypothesis 1*), and that they would be less likely and slower to orient from the eyes than the typically developing groups (*hypothesis 2*). We also hypothesized that WS would be associated with hypo-arousal during social perception compared to the typically developing groups (*hypothesis 3*). We tested this hypothesis by comparing *peak saccade velocity* between WS individuals and typically developing controls. The peak of the saccadic velocity is a sensitive measure of tonic arousal, which is reduced in individuals with conditions causing low tonic arousal, as well as in healthy participants after arousal- reducing manipulations such as sleep deprivation or pharmacological manipulations targeting the arousal systems of the brain (Di Stasi, Catena, Cã Nas C, Macknik, & Martinez-Conde, 2013).

We expected all groups to show a bias to attend to eyes, manifesting as (1) shorter latencies for gaze shifts from mouth to eyes than from eyes to mouth; (2) greater likelihood of gaze shifts from mouth to eyes than from eyes to mouth. We further hypothesized that typically developing individuals would show an increased bias to attend to eyes with increasing age, but that this developmental change would not be seen in WS. Stimulus images of actors displaying either a happy, angry, or neutral expression were included to examine whether the expected results generalize across emotional expressions.

*Pre-registration*.

The hypotheses and analysis plan were pre-registered prior to data analysis [link: https://osf.io/h3xp6/]

**Methods**.

*Participants*.

*Individuals with WS* (final *n =* 37; mean age = 23 years, SD = 12) age range 9–53) were recruited through the Center for Rare Diseases at Karolinska University hospital, Ågrenska – a national centre of competence for rare diseases in Sweden and through contact with patient organizations, and through contact with patient organizations. All participants had been diagnosed with WS in specialized health care. Since many individuals were diagnosed early by *Fluorescent in situ hybridization* (FISH) there was no information about the size of the deletion available in most adults. The individuals included in this study are all part of a larger ongoing study about WS. To date, 24/41 included individuals included in our study have been extensively investigated. Peripheral blood samples, urine, and skin sampling have been conducted to determine standard routine blood parameters and to detect endocrine abnormalities, hypercalcemia, and impaired glucose tolerance and fibroblasts (12 individuals) have been taken for DNA analyses, RNA-based studies and for the establishment of an iPSC-NE model for WS (4 individuals) to reveal mechanisms behind neural dysfunction. The 24 individuals with WS also completed an extensive clinical investigation, a cognitive assessment, and a diagnostic interview for anxiety disorders with a clinical psychologist. IQ was assessed using the Wechsler Adult Intelligence Scale, 4th Ed. (WAIS-IV; Wechsler, 2008) or the Wechsler Intelligence Scale for Children (WISC-5; Wechsler, 2014), depending on the participant’s age. The remaining individuals (*n* = 18) were tested in a temporary lab facility but did due to Covid-19 not yet complete additional assessments. In addition, 5/41 individuals were excluded because of a lack of valid data (see *Data Processing*). The WS participants were not split into age groups due to limited sample size.

*Typically developing infants* (final *n* = 37; mean age 7 months, SD = 0.20 months) were recruited from a database of families expressing interest in developmental research. Exclusion criteria were known or suspected medical or developmental concern, premature birth, or a family history of genetic syndromes or neurodevelopmental disorders up to second degree relatives. Two additional infants were excluded because of a lack of valid data (see *Data Processing*).

*Typically developing children and adolescents* (final *n* = 80, age 10–17 years) completed the task as part of control groups for two other studies. The first group (*n* = 39, mean age 12 years, SD = 2.64) was initially recruited from a database of families expressing interest in participating in developmental research and included as a control group in studies of ADHD. Details about the sample are reported in previous publications see (Frick, Brocki, Henriksson & Kleberg, 2022; Kleberg, Frick & Brocki, 2020) . The second group (*n* = 41; mean age 14 years, SD = 2.43) were recruited from the Swedish tax registry (for details about the sample, see (Kleberg et al, 2021a; Kleberg et al, 2021b) and initially included as a control group in a study on social anxiety disorder. Participants had no medical or psychiatric diagnoses according to parental report. In the second group, this was confirmed through a diagnostic interview with a clinical psychologist using the MINI-KID (Sheehan et al., 2010). For analytic purposes, the child sample was split into two age groups (8–12 and 13–17 years old, henceforth child and adolescent groups respectively).

*Typically developed adults* (*n =* 50; mean age 34 years; SD = 11) were recruited through a database of individuals who had expressed interest in participating in research experiments. The typically developed adults had no current medical diagnoses and were not taking any psychotropic medication. A semi-structured psychiatric interview was conducted with a clinical psychologist using the Mini International Neuropsychiatric Interview (MINI; Sheehan et al., 1998).

Age, gender, and average number of valid trials per group are shown in Table 1. The WS and infant groups completed a smaller number of valid trials than adults and adolescents in the eyes primed condition (*p* < .01), and a smaller number of trials than typically developing children, adolescents, and adults in the mouth primed condition (*p* < .01). As can be seen in Table 1, the gender proportion was uneven in the child and adolescent control groups reflecting recruitment choices made in the original studies where these individuals were included. Preliminary analyses showed no effect of gender on any of the dependent variables (all *p > .*25), and we therefore chose to include all participants in these groups instead of making a stratified selection based on gender.

## Ethical approval

The study was approved by the Regional Ethics Committee of Stockholm, Sweden. The recruitment of the child and adolescent groups was conducted in accordance with ethical approvals by the Regional Ethics Committees of Stockholm, Sweden, and Uppsala, Sweden.

**Table 1:** Age, gender, and number of valid trials

	WS	TD Adults	TD Adolescents	TD Children	TD Infants
**N**	37	50	36	44	37
**% Female**	46%	48%	21%	71%	43%
**Age in years: M, (SD), [min-max]**	23.43 (12.24) [9–53]	34.17 (11.41) [18.65–62.79]	15.54 (1.25) [13.10–17.30]	10.67 (1.27) [8.25–12.80]	0.58 (0.02) [0.55–0.62]
**Valid trials: M, (SD), [min-max]**					
**Eyes cued**	18.99 (7.71) [6–30]	29.28 (1.26) [25–30]	28.72 (2.22) [22–30]	25.04 (6.42) [7–30]	15.59 (7.13) [5–28]
**Mouth cued**	19.92 (8.24) [6–30]	27.72 (2.56) [20–30]	25.61 (5.43) [8–30]	22.76 (6.45) [5–30]	18.64 (6.67) [5–30]

*TD = Typically developing/developed*

*Power Analysis*.

A power analysis was conducted using the package *simr* (Green & Macleod, 2016) in R (R Development Core Team, 2015). This analysis indicated that the study had above 80% power to detect small-to medium effect sizes in pairwise group comparisons (marginal f^2^ = 0.15).

*Data Recording*.

Data were recorded using Tobii corneal reflection eye trackers (Tobii Inc, Danderyd, Sweden) at a sample rate of 120 HZ in the WS, healthy adult, adolescent, and infant groups. Data from children were recorded at a sample rate of 60 HZ (*n =* 39), or 120 HZ (*n =* 41).

*Data Processing*.

Fixations and saccades were identified using an I-VT filter implemented in MATLAB with velocity threshold criterion set to 30 degrees per second. To measure peak saccade velocity, data were first up-sampled to 1200 HZ using a simple spline function, since, it has been shown that peak velocities can be estimated with adequate precision in data recorded at 120 HZ or above after up-sampling (Mack, Belfanti, & Schwarz, 2017). Data sampled at 60HZ were not included in the peak velocity analyses, since peak saccade velocity cannot be recovered at this sample rate, even after up-sampling (Mack et al., 2017). Individuals contributing fewer than five valid trials to a condition were excluded from further analyses of that condition.

*Experimental task*.

**Fig. 1:**
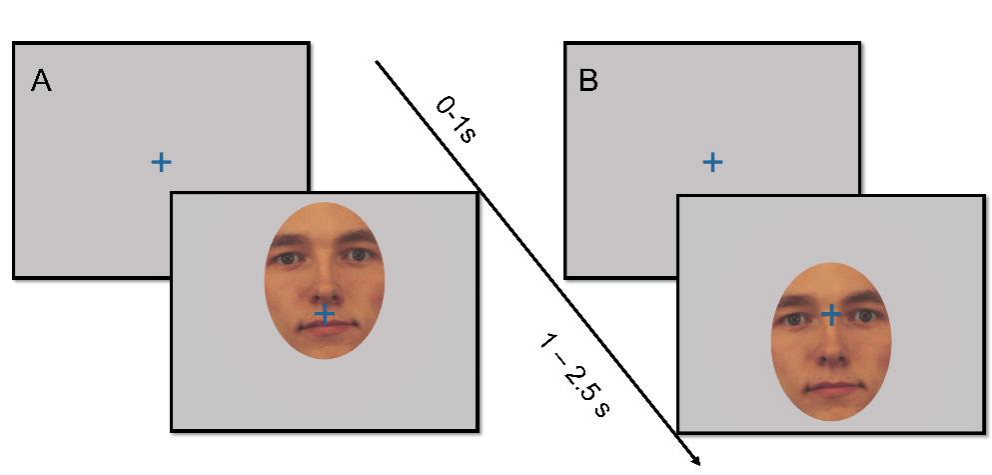
Overview of the experiment. In 50% of trials (A), a fixation cross presented for 1 s was followed by an image of a face with the mouth aligned to the fixation cross (the mouth cued condition) presented for 1.5 s. On 50% of the trials (B), the face was instead presented with the eyes in the position of the fixation cross (the eyes cued condition). The position of the fixation cross is shown overlaid on the face stimuli for illustrative purposes

Stimuli were images of human faces from the Karolinska Directed Emotional Faces dataset (Flykt, Lundqvist, Flykt, & Öhman, 1998), cropped to include only the inner region of the face (see Fig. 1). The depicted actors (5 male, 5 female) displayed an angry, happy, or neutral facial expression. Each actor appeared an equal number of times displaying each emotion. The experimental paradigm included 60 trials, equally distributed between two conditions. Trials began with a fixation cross presented on a uniform gray background for 1 s before the stimulus image appeared. In 50% of the trials, stimulus images were subsequently presented so that participants’ initial point of gaze was within the eye region (henceforth: the *eyes cued condition*). In the other 50% of the trials, stimuli were instead presented so that participants’ point of gaze was within the mouth (henceforth: the *mouth cued condition*). Stimulus images remained on screen for 1.5 s. Participants were asked to attend to the screen but were not given any further instructions and therefore this was a free viewing task.

*Dependent variables*.

The dependent variables were (1) the proportion of mouth-cued trials with a gaze shift towards the eyes; (2) the proportion of eyes-cued trials with a gaze shift away from the eyes; (3) the latency to orient to the eyes in the mouth-cued condition, and (4) the latency to orient away from the eyes in the eyes-cued condition; and (5) peak saccade velocity divided by saccadic amplitude to account for the fact that the distance and velocity of a saccade are typically closely correlated. All saccades with an amplitude exceeding 4 degrees detected during a trial were included in this metric, regardless of their direction. Preliminary analyses showed that the latency to orient to the non-cued facial region was highly correlated with the latency to orient from the cued region to any location (*r* = .86, *p* < .0001), indicating that first saccades landing outside the eye or mouth area were very rare. In line with previous studies (e.g. (Kleberg et al., 2021b; Kliemann, Dziobek, Hatri, Baudewig, & Heekeren, 2012), saccades landing outside any of the cued areas were therefore not included in the analyses.

*Statistical analyses*.

Data were analyzed using linear mixed-effects models with random intercept for participant (i.e., treating observations from the same individual as repeated measures). Initial omnibus models included the WS group (not split according to age) and typically developing individuals grouped according to chronological age. These analyses were followed up to address the specific hypotheses.

Statistical analyses were conducted in MATLAB using the functions *fitlme* and *compare*. Data were analyzed using both conventional frequentist (i.e., null hypothesis testing) and Bayesian statistics. Statistical significance was tested by comparing a model including the effect of interest (i.e., *group*) to the next most complex model (the null model) without this effect (Baayen, Davidson, & Bates, 2008). A Bayes factor (BF) was calculated from the Bayesian Information Criteria (BIC) values of the two models following Wagenmakers (2007) using the formula:

BF_10_ = exp ^(BIC_H0 −BIC_H1)/2^.

The BF quantifies the relative probability of the hypothesis (the model including the effect of interest) and the null (the same model without this effect). By convention, a BF > 3 indicates positive evidence for the hypothesis, a BF > 20 indicates strong support, and a BF > 150 very strong support (Wagenmakers, 2007). By reversing the terms, a BF_01_ can be calculated, where larger numbers indicate higher probability of the null. The main strengths of Bayesian as compared to frequentist statistics is that it is less vulnerable to type I errors, and that it allows researchers to conclude that the null hypothesis fits the data best (Wagenmakers, 2007). Effect sizes of the fixed effects are reported as unstandardized b coefficients and standardized marginal f^2^ (Nakagawa & Schielzeth, 2013), which reflect the proportional increase in explained variance that the fixed effect of interest contributes relative to the null model. The alpha level was set to 0.05. All significant results survived correction for multiple comparisons with the Benjamini-Hochberg procedure with a false discovery rate of 0.05. Uncorrected *p*-values are reported.

Results.

**Fig. 2:**
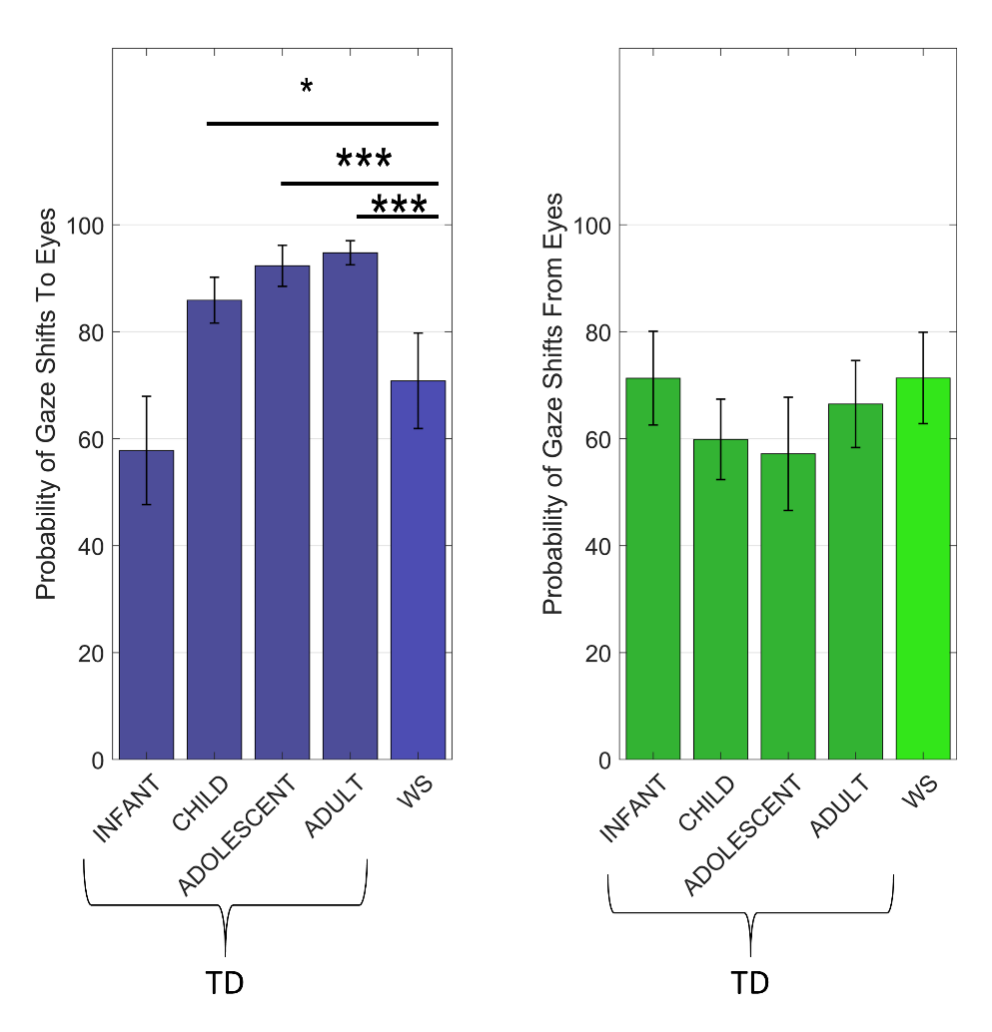
Probability of gaze shifts to the eyes (left) and from the eyes (right) in individuals with Williams syndrome (all ages) and typically developing individuals grouped by age range. WS = Williams syndrome; *** p < .001. Asterisks show significant difference between WS and the typically developing groups. Error bars show 95% confidence intervals

*Proportion of first gaze shifts.* An initial omnibus model including data from both WS and TD individuals of all age groups showed a significant effect of cued region, reflecting that the proportion of gaze shifts was higher to, than from the eyes (*χ* ^2^ = 27.55, *p* < .001, b = 0.16, SE = 0.03, BF_10_ > 500, f^2^ = 0.09). There was a main effect of group (*χ* ^2^ = 17.11, *p* = .002, BF_10_ = 397, f^2^ = 0.04), and an interaction effect between group and cued region (*χ* ^2^ = 44.13, *p* < .001, BF_10_ > 500, f^2^ = 0.27). Follow-up tests showed that groups differed in the proportion of gaze shifts to the eyes (*χ* ^2^ = 71.23, *p* < .001, BF_10_ > 500, f^2^ = 0.43; see Fig. 2). In contrast, no significant effect of group was found on the proportion of gaze shifts away from the eyes (*χ* ^2^ = 9.12, *p* = .058, f^2^ = 0.05), and the Bayesian analysis strongly supported the null hypothesis (BF_01_ = 401.17). Significant main- and interaction effects were followed up to address the specific hypotheses as described below.

*Is there a preference for eyes in typical development?* Gaze shifts towards the eyes were more likely than gaze shifts away from the eyes in the typically developing adult, adolescent, and child groups (all *p <* .001, all BF > 500). In contrast, typically developing infants showed the opposite pattern - i.e., a preference for orienting away from, rather than to the eyes (*χ* ^2^ = 4.18, *p* = .041, b = 0.13, SE = 0.06, BF_10_ = 1.53, f^2^ = 0.09).

*Does orienting to eyes change during typical development?* As can be seen in Table 2; Fig. 2, the probability of orienting towards the eyes increased in typically developing individuals from infancy to childhood, and from childhood to adolescence, but remained stable between adolescence and adulthood. The proportion of gaze shifts to the eyes was not related to age in the WS group (*χ* ^2^ = 0.03, *p* = .865, BF_10_ = 0.17, BF_01_ = 5.83, f^2^ < 0.01).

*Is there an altered preference for eyes in WS?* No significant difference in the proportion of gaze shifts to and from the eyes was found in the WS group (*χ* ^2^ = 0.01, *p* = .953, f^2^ < 0.01), and the Bayesian analysis supported the null hypothesis (BF_10_ = 0.16, BF_01_ = 6.23).

As can be seen in Table 3; Fig. 2, individuals with WS were less likely to orient to the eye region than typically developing children (*χ* ^2^ = 7.36, *p* = .007, BF_10_ = 4.25), adolescents (*χ* ^2^ = 13.06, *p* < .001, BF_10_ = 85.15), and typically developed adults (*χ* ^2^ = 27.08, *p* < .001, BF_10_ > 500). Individuals with WS were marginally more likely to orient to the eyes than infants (*χ* ^2^ = 4.23, *p* = .040, BF_10_ = 0.97), but the difference was not statistically significant after correction for multiple comparisons, and the Bayes factor was inconclusive.

*Does orienting to eyes change during development in WS?* The proportion of gaze shifts to the eyes was not related to age in the WS group (*χ* ^2^ = 0.07, *p* > .50, BF_10_ = 0.17, BF_01_ = 5.88, f^2^ < 0.01). The Bayes factors indicate that the data were 5.88 times more likely to occur under the null hypothesis, that is, providing evidence for the lack of an age effect in the WS group.

*Does emotional expression affect the probability to orient to eyes?* Individuals with WS were less likely than typically developed adults and adolescents to orient to the eyes of faces with each of the included emotional expressions (all *p* < .01, all BF_10_ > 15). Differences between the WS group and typically developing children were significant for happy and neutral, but not angry faces. No group differences were found between individuals with WS and typically developing infants (see *Supplementary materials*).

**Table 2:** Pairwise group comparisons of the probability of gaze shifts to the eyes. *** p < .001; **p < .01; † Non-significant after correction for multiple comparisons; WS = Williams syndrome; BF_*10*_ *= Bayes factor favoring the hypothesis; BF*_*01*_ = Bayes factor favoring the null. TD = Typically developing/developed

Group Comparison	Model comparison χ2	p	b	SE	BF_10_	BF_01_	f^2^
WS vs. TD Infant	4.23	0.040†	-0.14	0.07	0.97	1.03	0.06
WS vs. TD Child	7.36	0.007**	0.13	0.05	4.25	0.24	0.09
WS vs. TD Adolescent	13.06	< 0.001***	0.20	0.05	85.15	0.01	0.22
WS vs. TD Adult	27.08	< 0.001***	0.22	0.04	> 500	< 0.01	0.37
Infant vs. TD Child.	23.09	< 0.001***	0.27	0.05	> 500	< 0.01	0.31
TD Child vs. TD Adolescent	3.78	0.052	0.07	0.04	0.75	1.33	0.05
TD Adolescent vs. TD Adult	0.64	0.425	0.02	0.03	0.16	6.43	0.01

*Latency to first gaze shifts.* Results from the analyses of saccadic latencies mirrored the findings from the analyses of proportion of first gaze shifts. An initial omnibus model including data from both WS, and TD individuals showed a significant effect of cued region, reflecting quicker gaze shifts towards the eyes than away from the eyes (*χ* ^2^ = 46.99, *p* < .001, b = 199, SE = 27, BF_10_ > 500, f^2^ = 0.07). There was also a main effect of group (*χ* ^2^ = 90.90, *p* < .001, BF_10_ > 500, f^2^ = 0.08), and an interaction effect between group and cued region (*χ* ^2^ = 27.86, *p* < .001, BF_10_ = 28.03, f^2^ = 0.04).

Follow-up tests showed that gaze shifts were quicker to the eyes than away from the eyes in typically developing adults (*χ* ^2^ = 32.27, *p* < .001, BF_10_ > 500, b = 349, SE = 52, f^2^ = 0.27), adolescents (*χ* ^2^ = 21.88, *p* < 001, BF_10_ > 500, b = 314, SE = 56, f^2^ = 0.35) and children (*χ* ^2^ = 22.93, *p* < .001, BF_10_ > 500, b = 233, SE = 43, f^2^ = 0.12). In contrast, no consistent preference was found in WS (*χ* ^2^ = 0.23, *p* > .50, BF_10_ = 0.19, b = 37, SE = 76, f^2^ < 0.01) or typically developing infants (*χ* ^2^ = 0.19, *p* > .50, *b* = 23, *SE* = 54, BF_10_ = 0.19, f^2^ < 0.01). In typically developing individuals, the latency of first gaze shifts towards the eyes decreased from infancy to childhood, and again from childhood to adolescence, but remained stable between adolescence and adulthood.

**Fig. 3:**
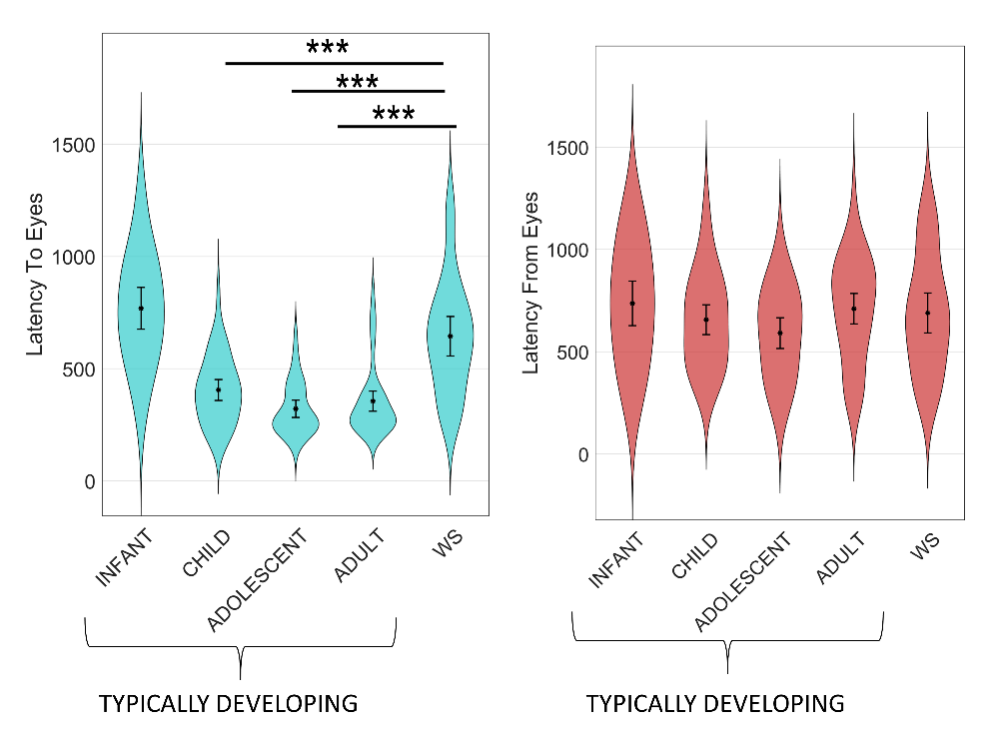
Latencies to orient to eyes (left) and from eyes (right) in individuals with Williams syndrome (all ages) and typically developing individuals grouped by age range. Error bars show 95% confidence intervals. WS = Williams syndrome; TD = Typically developing/developed; *** p < .001. Violins show probability density functions. Asterisks show significant difference between WS and other groups. Error bars show 95% confidence intervals of the mean

Individuals with WS were slower to orient to the eye region than typically developing children, adolescents, and adults. They were marginally faster than typically developing infants, but the p-value did not survive correction for multiple comparisons, and the Bayes factor was inconclusive (see Table 3; Fig. 3). The latency to orient to the eyes was not linked to age in the WS group (*χ* ^2^ < 0.01, *p* = .969, b = 0.14, SE = 3.55, BF_10_ = 0.18, BF_01_ = 5.65), and the Bayes factor supported the null hypothesis, i.e., the lack of an age effect. Additional analyses showed that the WS group oriented slower than typically developing adults, adolescents, and children to the eyes of faces with each of the included emotional expressions (all *p* < .001, all BF_10_ > 500, see *Supplementary materials*).

To sum up, contrary hypothesis 1, individuals with WS were both *less likely* and *slower* to orient to eyes than typically developing controls, and contrary to hypothesis 2, did not differ from controls in the probability or latency to orient from the eyes. Whereas the expected preference to attend to eyes rather than the mouths was found in typically developing individuals above infancy, this bias was absent in individuals with WS.

**Table 3:** Pairwise group comparisons of the latency to first gaze shift to the eyes. *** p < .001; * p < .05; † Non-significant after correction for multiple comparisons; WS = Williams syndrome; BF_10_ = Bayes factor favoring the hypothesis; BF_01_ = Bayes factor favoring the null. TD = Typically developing/developed

Group Comparison	χ2	P	b	SE	BF_10_	BF_01_	f^2^
WS vs. TD Infant	4.12	0.042†	-132.35	63.85	1.01	0.99	0.03
WS vs. TD Child	25.35	< 0.001***	236.28	43.71	> 500	> 01	0.12
WS vs. TD Adolescent	31.63	< 0.001***	318.93	49.69	> 500	> 01	0.28
WS vs. TD Adult	29.17	< 0.001***	269.93	45.92	> 500	> 01	0.18
TD Infant vs. TD Child.	47.94	< 0.001***	366.88	45.26	> 500	> 01	0.23
TD Child vs. TD Adolescent	5.87	0.015*	79.38	32.14	2.17	0.46	0.02
TD Adolescent vs. TD Adult	1.80	0.180	49.34	36.60	0.28	3.59	< 0.01

*Is arousal reduced in WS during social perception?*

Peak saccadic velocity was lower in the WS group (*M* = 22.45, *SD =* 5.85) than in typically developing adults (*M =* 29.67, SD = 4.14), adolescents (*M* = 29.38, SD = 4.56), and infants (*M =* 25.36, *SD =* 7.73), reflected in a main effect of group, *χ* ^2^ = 84.82, *p* < .001, BF_10_ > 500, f^2^ = 0.28). There was also a main effect of condition, reflecting higher peak saccadic velocity when the mouth was primed (*χ* ^2^ = 11.13, *p* = .001, BF_10_ = 18.29, f^2^ = 0.01, M_mouth_ = 27.37, SD_mouth_ = 4.85, M_eyes_ = 26.74, SD_eyes_ = 5.06). No interaction effect between group and condition was found (*χ* ^2^ = 5.95, *p* = .203, BF_10_ < 0.01, BF_01_ > 500, f^2^ < 0.01. As can be seen in Table 4, the WS group had reduced peak saccadic velocity, indicating reduced arousal, compared to typically developing individuals in all age ranges. In the WS group, no relations were found between peak saccadic velocity and the latency to orient from the eyes, (*χ* ^2^ = 0.08, *p* > .50, BF_10_ = 0.17, BF_01_ = 5.76, f^2^ = 0.01), or the proportion of gaze shifts from the eyes (*χ* ^2^ = 0.66, *p* = .418, BF_10_ = 0.23, BF_01_ = 4.38, f^2^ = 0.01), with Bayes factors favoring the null.

**Table 4:** Pairwise group comparisons of peak saccadic velocity (divided by saccadic amplitude). *** p < .001; ** p < .01; WS = Williams syndrome; BF_10_ = Bayes factor favoring the hypothesis; BF_01_ = Bayes factor favoring the null. TD = Typically developing/developed

Group Comparison	χ2	P	b	SE	BF_10_	BF_01_	f^2^
WS vs. TD Infant	8.28	0.004**	3.24	1.09	7.30	0.14	0.05
WS vs. TD Child	34.10	< 0.001***	7.65	1.09	> 500	< 0.01	0.49
WS vs. TD Adolescent	42.84	< 0.001***	6.97	0.89	> 500	< 0.01	0.37
WS vs. TD Adult	79.29	< 0.001***	7.31	0.64	> 500	< 0.01	0.49
Infant vs. TD Child.	7.58	.006*’	4.44	1.55	4.69	0.21	0.12
Child vs. TD Adolescent	0.48	0.487	-0.70	1.01	0.14	7.03	< 0.01
Adolescent vs. TD Adult	0.30	0.584	0.35	0.63	0.13	7.60	< 0.01

Discussion.

In line with the commonly observed hyper-social behavioral phenotype associated with WS, we hypothesized that individuals with the condition would show increased attention to others’ eyes, manifesting as (1) an increased tendency to orient to the eye region of others, and (2) prolonged attention to the eyes of others once they were fixated. These hypotheses were not supported. Instead, *reduced* orienting to other’s eyes was found in individuals with WS. Whereas typically developing individuals from eight years old to adulthood showed the expected bias to orient to eyes rather than to the mouth, this bias was absent in the WS group, with no evidence for age effects. Furthermore, individuals with WS were slower and less likely to orient to the eye region than typically developing individuals across a wide age range. Notably, reduced orienting to eyes was found for all emotional expressions. Finally, we hypothesized that individuals with WS would show reduced arousal during social perception. This hypothesis was supported in that individuals with WS showed reduced peak saccadic velocity compared to all typically developing age groups from infancy to adulthood. To the best of our knowledge, this is the largest eye tracking study of WS to date (*n* = 39). The conclusions were supported by both Bayesian and frequentist statistics, with medium to strong effect sizes. The results are discussed below.

A largely subcortical brain network including the amygdala supports detection of eye contact and orienting towards others’ eyes (Johnson et al., 2015). Our results suggest that this network is atypical in WS. At the behavioral level, our results suggest that, at the earliest time stages of visual attention, WS is associated with reduced, rather than enhanced attention shifts to eyes, which are arguably one of the most salient social stimuli for humans. This is a striking difference to the results from studies examining how individuals with WS attend to eyes or faces presented for longer time periods ranging from several seconds to minutes. These studies have generally reported prolonged attention to eyes (Mervis et al., 2003; Riby & Hancock, 2008) or whole faces presented among non-social distractors (D’Souza et al., 2015; Doherty-Sneddon et al., 2009; Mervis et al., 2003; Riby & Hancock, 2008, 2009), and have assessed attention by accumulating looking time over the entire stimulus presentation period. Our results suggest that social attention alterations observed in WS are dependent on the time stage at which they are studied. This points to the importance of measuring social attention using high spatial and temporal resolution in studies of WS and other neurodevelopmental disorders. It should be noted that previous studies have not cued participant’s gaze to specific areas within the face, and have generally examined social attention in situations where social stimuli compete for attention with various non-social distractors.

The eye region conveys crucial information about other’s mental states and their intentions regarding the environment. However, decoding this information requires the ability to flexibly shift one’s attention between others’ eyes and the surroundings (del Bianco, Falck-Ytter, Thorup, & Gredebäck, 2019; Vivanti et al., 2017). Disruptions to these processes could contribute to the difficulties that many individuals with WS face within areas such as mental state understanding and gaze following. In a study assessing autistic symptoms using the ADOS in young children with WS, 50% of participants were described as having “unusual eye contact” (Klein-Tasman, Phillips, Lord, Mervis, & Gallo, 2009).

Individuals with WS commonly show domain-general attention impairments, including difficulties with attention shifting (Atkinson et al., 2007; Breckenridge et al., 2013). Our results are not readily explained by such a domain-general impairment in attention shifting. A typical sensitivity to eyes in WS participants would be expected to result in a bias to orient to the eyes rather than the mouth, even if both types of gaze shifts would be slower than in typically developing participants because of difficulties with attention shifting. Instead, WS participants did not show a bias to orient to the eyes over the mouth, a null result which was strongly supported by the Bayesian analysis, and which was found despite adequate statistical power. It should be noted that difficulties with disengagement of attention could still explain other findings in the literature, such as increased attention to faces presented among other visual stimuli or during face to face interaction (Dodd & Porter, 2010; Doherty-Sneddon et al., 2009; Riby & Hancock, 2009).

Previous studies have suggested that tonic hypo-arousal is a factor contributing to enhanced social attention in WS. Consistent with previous studies, we found that individuals with WS executed saccades with reduced peak amplitude (indicating reduced arousal) compared to all the TD groups, including seven-month-old infants (Doherty-Sneddon et al., 2009; Riby et al., 2012; Skwerer et al., 2009). However, the hypothesized link between hypo-arousal and *enhanced* eye contact was not supported, as the WS group did not attend longer to the eye region before reorienting, and individuals with relatively lower peak saccadic velocity within the WS group did not take longer to reorient from eyes. Instead, hypo-arousal could be an underlying cause of reduced orienting to eyes. It is known from previous studies that hypo-arousal can impair detection and orienting of attention to salient and motivationally important stimuli (Kleberg, Frick, & Brocki, 2020; Petersen & Posner, 2012). We suggest that hypo-arousal may have different effects on social attention depending on the stage of visual attention. Hypo-arousal may facilitate prolonged social attention once it is established (Doherty-Sneddon et al., 2009), but also reduce the likelihood that it is established.

Our results point to a potential overlap between WS and ASC, which is associated with reduced orienting to social stimuli such as faces and eyes (Hedger, Dubey, & Chakrabarti, 2020; Kleberg et al., 2017)^,^. This similarity is interesting, given the fact that WS is also associated with increased social motivation and interest in faces, traits that are not typically associated with ASC (Barak & Feng, 2016). Previous studies comparing visual attention in WS and ASC have also suggested that individuals with WS attend longer to whole faces than those with ASC, although not examining eyes specifically (Riby & Hancock, 2008, 2009). Our results suggest that social interaction challenges in both conditions may be associated with a reduced ability to process eye gaze. Future studies should examine how the observed results relate to within-syndrome variability in WS, by examining potential links to co-occurring autistic traits and anxiety, which are frequently associated with the condition.

Altered visual orienting to eyes in WS was not modulated by the emotional expression of the face. In fact, WS individuals showed a highly typical pattern of increased attention to the eyes of angry faces and the mouth of happy faces. This contrasts with previous studies which reported atypical attention to (whole) faces signaling positive emotional states or trustworthiness in WS (Boulton & Porter, 2020; Dodd & Porter, 2010).

The inclusion of multiple comparison groups across a wide range enabled us to examine how individuals with WS deviate from the typical developmental trajectory in considerable detail. However, a limitation is that the WS group did not include individuals in infancy and toddlerhood, which reduces our ability to detect developmental trajectories in this population. A second limitation is that arousal was only assessed through the saccadic velocity profile, which has previously not been used in studies of WS. Future studies should ideally combine this measure with other indices of physiological arousal, such as skin conductance, and use eye trackers with higher sampling rates to enable measurement of saccadic velocity profiles without up-sampling. Future studies would also benefit from comparisons between WS individuals and groups with other genetic syndromes affecting attention, cognition, and social behavior.

To sum up, this study suggests that despite the highly social behavioral phenotype, WS is associated with reduced orienting to other’s eyes at the earliest time stages of attention. Hypo-arousal during social perception may contribute to this pattern of atypical attention. Disrupted orienting to other’s eyes is likely to affect social understanding and interaction. These findings add to our understanding of the pathways from genetic deletions to altered social phenotype in WS.
